# Apolipoprotein ε genotype and sex influences skeletal muscle bioenergetic response to diet

**DOI:** 10.1002/alz.086251

**Published:** 2025-01-03

**Authors:** Chelsea N Johnson, Colton R Lysaker, Colin S McCoin, Riley E Kemna, Casey S. John, John P Thyfault, Heather M Wilkins, Paige C Geiger, Jill K Morris

**Affiliations:** ^1^ University of Kansas Alzheimer’s Disease Research Center, Fairway, KS USA; ^2^ University of Kansas Medical Center, Kansas City, KS USA

## Abstract

**Background:**

Apolipoprotein ε4 (*APOE4*) is a major risk factor for Alzheimer’s disease (AD). *APOE4* carriers display altered whole‐body metabolism, including increased blood glucose and inuslin. Although conditions affecting whole‐body metabolism like obesity and diabetes are AD risk factors, knowledge regarding the contribution of peripheral tissues to this effect is minimal. Skeletal muscle is an important regulator of whole‐body metabolism as a primary site for nutrient oxidation, yet it is unclear if *APOE4* affects muscle metabolism. We sought to determine the impact of *APOE4* on muscle bioenergetic function.

**Method:**

Male and female *APOE3* and *APOE4* targeted‐replacement mice on a C57BL/6 background (n = 61) from Taconic were fed a high (HFD, 45% fat) or low‐fat diet (LFD, 10% fat) for 4 months before sacrifice. *APOE3* mice served as controls because *APOE3* does not modify AD risk. At 8‐9 months old, carbohydrate‐driven oxygen consumption in isolated quadriceps mitochondria was measured at basal, state 3 (ADP), state 3 + glutamate, and state 3S (succinate) on the Oroboros Oxygraph‐2k and the gastrocnemius proteome was analyzed on a timsTOF HT mass spectrometer. Mitochondrial‐related metabolic proteins were identified using MitoCarta3.0. Serum lactate was measured using an Abcam kit.

**Result:**

*APOE4* associated with differential expression of 49 proteins compared to *APOE3* in LFD females only (q‐value <0.05), including reduced levels of proteins involved in oxidative phosphorylation. *APOE4* did not affect mitochondrial respiration. HFD altered the expression of 469 proteins in *APOE4* females and between 80‐101 proteins in other groups (q‐value <0.05). HFD upregulated proteins involved in lipid metabolism in all groups. HFD upregulated proteins involved in carbohydrate metabolism and increased mitochondrial respiration (basal, p = 0.062; state 3, p = 0.009; state 3 +glutamate, p = 0.032; state 3S, p = 0.17) in *APOE4* females only. HFD reduced serum lactate in *APOE3* males (p = 0.041), *APOE4* males (p = 0.081) and *APOE3* females (p = 0.112).

**Conclusion:**

HFD‐driven upregulation of proteins involved in carbohydrate metabolism in *APOE4* female muscle only likely supports increased carbohydrate oxidative capacity and may explain the lack of reduction in serum lactate in this group. Proteomic analysis of human muscle is ongoing to determine if this is reflected in humans.

